# Novel Targets in a High-Altitude Pulmonary Hypertension Rat Model Based on RNA-seq and Proteomics

**DOI:** 10.3389/fmed.2021.742436

**Published:** 2021-11-03

**Authors:** Xiang Xu, Hanlu Li, Qingxia Wei, Xin Li, Yanying Shen, Ge Guo, Yibing Chen, Kunlun He, Chunlei Liu

**Affiliations:** ^1^Laboratory of Translational Medicine, Medical Innovation Research Division of Chinese PLA General Hospital, Beijing, China; ^2^Beijing Key Laboratory of Chronic Heart Failure Precision Medicine, Chinese PLA General Hospital, Beijing, China

**Keywords:** high-altitude pulmonary hypertension, selexipag, macitentan, RNA-seq, proteomics, reoxygenation

## Abstract

High-altitude pulmonary hypertension (HAPH) is a complication arising from an inability to acclimatize to high altitude and is associated with high morbidity and mortality. We aimed to analyze the effects of macitentan, selexipag, riociguat, and reoxygenation on HAPH, and to screen possible targets of these treatments for future drug screening. Rats were subjected to hypobaric hypoxia for 35 days to induce HAPH, and treated with vehicle or selexipag, macitentan, riociguat, or with reoxygenation, from days 21 to 35. Selexipag, macitentan, and reoxygenation prevented an increase in mean pulmonary artery pressure and hypoxia-induced right ventricular hypertrophy, compared to the vehicle. Riociguat had little effect. RNA-seq and proteomics revealed strong correlations between responses to the three drugs, which had almost identical effects. GO-enrichment revealed that the differentially expressed genes included those involved in metabolic regulation, transcription, and translation. Various molecular pathways were annotated. Selexipag, macitentan, and reoxygenation ameliorated HAPH. Serpina1, Cryz, and Cmc1 were identified, *via* multi-omics screening, as key genes involved in HAPH. These findings provide new insights into the targeted drug mechanisms in HAPH.

## Introduction

Pulmonary hypertension (PH) refers to a resting mean pulmonary artery pressure ≥25 mmHg. It is divided into five categories, based on hemodynamic characteristics, pathogenesis, and pathology (1). High-altitude pulmonary hypertension (HAPH), in the third category, is caused by a compensatory increase in lung ventilation and pulmonary arteriole vasoconstriction in a high-altitude hypoxic environment and leads to high altitude-induced cardiomyopathy. The clinical symptoms include exercise dyspnea, headache, and fatigue. HAPH may reflect a failure to acclimatize to high altitude, accompanied by unclear pathophysiological mechanisms. About 140 million people live at >2,500 m above sea level, and >40 million people visit these high-altitude regions each year (2, 3). Hypoxia causes the gene expression profiles of organs to change differentially; this is known from adaptive changes in the genotype of long-term high-altitude residents and their offspring (4).

Treatments for HAPH are still being investigated. Patients should be advised to move to lower altitude, and oxygen therapy is effective (5). Studies of drug treatment are limited to some randomized trials (6). HAPH has similar pathology to other types of PH, so it is worth considering whether drugs that are effective for PAH might be effective for HAPH. In addition to conventional drugs, such as vasodilators, targeted drugs have been increasingly studied. Activation of endothelin receptor 1 leads to pulmonary vasoconstriction and smooth-muscle cell proliferation. Macitentan is a dual antagonist of the endothelin receptor, with enhanced penetration and low risk of hepatotoxicity when administered for pulmonary arterial hypertension (7–10). Prostacyclin is released by endothelial cells and promotes pulmonary vasodilation, with antithrombotic and antiproliferative effects. Selexipag, an oral prostacyclin (PGl2) receptor agonist, differing in structure from prostacyclin, has been reported to be more effective than placebo in reducing morbidity and mortality in patients with PAH (11, 12). Endothelial nitric oxide (NO) production was lower and phosphodiesterase type 5 expression was higher in pulmonary artery smooth-muscle cells and in the right ventricular myocardium in patients with PAH than in those without PH (13–15). NO activates soluble guanylate cyclase (sGC), stimulating cyclic guanosine monophosphate (cGMP) production, leading to vasodilation of small arteries, and inhibiting cell proliferation. Further, phosphodiesterase type 5 hydrolyzes cGMP. Riociguat is a soluble guanylate cyclase stimulator, and can promote vascular remodeling and pulmonary vasodilation without depending on NO. Riociguat increases the sensitivity of sGC to NO, thus raising cGMP levels (16, 17).

However, all of these targeted drugs have been studied in patients with symptomatic PH, whose pathogenesis was idiopathic, familial, and was associated with connective-tissue disease, portal hypertension with liver cirrhosis, or toxin exposure. Almost no HAPH patients have been included in these studies. Therefore, the effectiveness and mechanisms of these drugs in HAPH remain unclear. To address this, we aimed to examine the molecular mechanisms involved in hypoxia, using a rat HAPH model, and applying an integrated multi-omics approach. In doing so, we aimed to investigate the effects of several targeted drugs and reoxygenation to identify potential new therapeutic targets for altitude-induced hypoxia. In particular, we examined how the targeted drugs affected differential gene and protein expression among organs.

## Materials and Methods

### Animals

Sprague-Dawley rats (male, 220–250 g) were purchased from the Animal Experiment Center of the Chinese PLA General Hospital (Beijing, China). All animal experimental procedures were approved by the Animal Ethics Committee of the Chinese PLA General Hospital (approval number: 2017-X13-05). The animals were raised under a 12 h light/dark cycle, with free access to food and water. Room temperature was 22–25°C. Bedding was changed twice a week. Control group rats were housed in a normoxia environment and treated with vehicle after 3 weeks. The remaining animals were randomly assigned to six groups at 3-week time point, one group was sacrificed for assessing the cardiopulmonary function on day 21, the other five groups were M (model group): chronic hypoxia, vehicle-treated; PGI2Y: selexipag-treated (5 mg/kg, bid.); ETAY:macitentan-treated (30 mg/kg, q.d.); SGCY: riociguat-treated (10 mg/kg, q.d.); and RE: reoxygenation-treated. Except for group C, the other five group rats were housed in a hypobaric hypoxia chamber for 35 d; treatments started on day 21 and continued for 2 weeks. Each group had 6 rats. We regarded the reoxygenated group as the positive control group. Selexipag, macitentan, and riociguat were purchased from Selleck Chemicals (catalog numbers S3726, S8051, and S8135; USA).

### Chronic Hypoxia-Induced PH and Drug Treatment

The rats were placed in a 10% O_2_ (hypoxic) chamber for 3 weeks to develop PH. Control rats (group C) were housed under normoxia for the entire experiment and treated with the vehicle during the treatment period (i.e., from day 21). At 3 weeks, the remaining rats were randomized into six groups; one group was sacrificed (day 21) to assess cardiopulmonary function. For euthanasia, animals were anesthetized by pentobarbital sodium (90 mg/kg body weight), and mean pulmonary arterial pressure (mPAP) was calculated to validate the model. The remaining five groups were treated with targeted drugs or reoxygenation, from day 21 to sacrifice on day 35. These groups were; chronic hypoxia, vehicle-treated (M); chronic hypoxia, selexipag-treated (5 mg/kg, bid.) (PGI2Y); chronic hypoxia, macitentan-treated (30 mg/kg, q.d.) (ETAY); chronic hypoxia, riociguat-treated (10 mg/kg, q.d.) (SGCY); chronic hypoxia, reoxygenation (RE). Selexipag, macitentan, and riociguat were purchased from Selleck Chemicals (catalog numbers S3726, S8051, and S8135; USA).

### Hemodynamic Measurements and Sample Collection

Vascular pressure was assessed using Millar catheters, as previously described (18). Rats were fixed on the operating table, anesthetized, tracheotomized, and placed on ventilator-assisted breathing (Kent Scientific, USA). A Millar SPR 838 pressure–volume catheter (ADInstruments, USA) was inserted through a parasternal incision into the right ventricle (RV), then advanced into the pulmonary artery. Pressure measurements were acquired using an MPVS Ultra system coupled to a PowerLab data acquisition system (ADInstruments) to calculate mPAP.

### Sample Preparation and Assessment of RV Hypertrophy

After catheterization and measurements, the lungs and heart were harvested, and washed twice with ice-cold saline to remove blood and other contaminants. The RV and left ventricle (LV) with septum (LV + IVS) were weighed, and their mass ratio, the RV hypertrophy index, RVHI = RV/(LV + IVS), was calculated. The upper left lung was fixed by inflation with 10% formalin, embedded in paraffin, and sectioned for histology.

### RNA-seq Analysis

#### RNA Extraction and Qualification

RNA from the samples was extracted using TRIZOL (1 mL/200 mg, Life Technologies, Rockville, MD) according to the manufacturer's protocol. RNA quality and concentration were checked using a NanoDrop 2000 spectrophotometer (Thermo Fisher Scientific, Wilmington, DE). Purified RNA was stored at −80°C until required. RNA samples were reverse-transcribed using the Reverse Transcription System (Promega, USA).

#### RNA-seq and Computational Analysis

Total RNA was extracted, and mRNA and noncoding RNAs were enriched by depleting rRNA using an Arraystar rRNA Removal Kit. The mRNAs and noncoding RNAs were broken into short fragments (~200–500 nt) by the fragmentation buffer. The short fragments were used as templates, and first-strand cDNA was synthesized using random hexamer primers. dTTP was replaced by dUTP during second-strand synthesis. Elution buffer was then added to purify and resolve the short fragments *via* end-repair and addition of adenine. Short fragments were purified and connected with adaptors; the second strand was digested using uracil-N-glycosylase (19). After agarose gel electrophoresis, suitable fragments were selected as templates for PCR amplification. Quantification and quality assessment of the sample library were performed using an Agilent 2100 Bioanalyzer and ABI StepOnePlus Real-Time PCR System. The library was sequenced using an Illumina HiSeq 2000 system.

Protein concentration was determined *via* Bradford Protein Assay. The samples were analyzed on a Q Exactive HF mass spectrometer (Thermo Fisher Scientific) interfaced with an Easy-nLC 1000 nanoflow LC system (Thermo Fisher Scientific). For each sample, 4 μL of digested protein was loaded onto a Biosphere C18 Precolumn (2 cm × 100 μm; particle size, 3 μm; pore size, 300 Å) at 7.5 μL/min. After 3 min, the protein samples were separated using a 150 μm × 12 cm silica microcolumn (homemade; particle size, 1.9 μm; pore size, 120 Å) with a linear gradient of 5–35% mobile phase B (0.1% formic acid in acetonitrile) at a flow rate of 600 nL/min for 75 min. Using a data-dependent strategy, by measuring MS1 in an Orbitrap mass spectrometer with a resolution of 120,000, and then using high-energy collision dissociation with a normalized collision energy of 27% and a dynamic rejection time of 18 s, the first 20 precursors were subjected to tandem mass spectrometry. Trypsin digestion of 293T cells was used for routine quality control of samples, to ensure sensitivity and repeatability.

#### Data Preprocessing

Transcripts with fragments per kilobase of transcript per million mapped reads (FPKM) >0.1 in at least 20% of the samples were retained for NCBI omics-database screening. Sequence expression was subsequently normalized using quantiles (20). For the logistic regression analysis, feature expression was further normalized to a normal distribution using the z-score algorithm (21).

### Proteomic Measurement

#### Protein Trypsin Digestion

Whole-tissue protein extractions: 0.1 g tissues were lysed with 400 μl urea lysis buffer (8 M urea, 100 mM Tris-HCl pH 8.0), 4 μl protease inhibitor (PierceTM, Thermo Fisher Scientific) was added to protect protein from degradation and protein concentrations were measured using Bradford method (Eppendorf Biospectrometer). One hundred micrograms of proteins were digested by FASP procedure. Namely, the protein samples were supplemented with 1 M dithiothreitol (DTT) to a final concentration of 5 mM and incubated for 30min at 56°C, then added iodoacetamide (IAA) to a 20 mM final concentration, and incubated in the dark at room temperature. After half an hour incubation, samples were added 5 mM final concentration of DTT and keep in dark for another 15 min. After these procedures, protein samples were loaded into 10 kD Microcon filtration devices (Millipore) and centrifuged at 12,000 g for 20 min and washed twice with Urea lysis buffer (8 M Urea, 100 mM Tris-HCl pH8.0), twice with 50 mM NH_4_HCO_3_. Then the samples were digested using trypsin at an enzyme to protein mass ratio of 1:25 overnight at 37°C. Peptides were extracted and dried (SpeedVac, Eppendorf).

#### LC-MS/MS Analysis

Samples were analyzed on a Q Exactive HF mass spectrometer (Thermo Fisher Scientific, Rockford, IL, USA) connected to an Easy-nLC 1000 liquid chromatography system (Thermo Fisher Scientific). Dried peptide samples were re-dissolved in Solvent A (0.1% formic acid in water) and loaded to a trap column (100 μm × 2 cm, homemade; particle size, 3 μm; pore size, 120 Å; SunChrom, USA) with a max pressure of 280 bar using Solvent A, then separated on a home-made 150 μm × 30 cm silica microcolumn (particle size, 1.9 μm; pore size, 120 Å; Dr. Maisch GmbH) with a gradient of 5–35% mobile phase B (acetonitrile and 0.1% formic acid) at a flow rate of 600 nl/min for 150 min. The MS analysis for QE HF was performed with one full scan (300–1,400 m/z, *R* = 120,000 at 200 m/z) at automatic gain control target of 3e6 ions, followed by up to 30 data-dependent MS/MS scans with higher-energy collision dissociation (target 2e4 ions, max injection time 40 ms, isolation window 1.6 m/z, normalized collision energy of 27%), detected in the Orbitrap (*R* = 15,000 at 200 m/z). The dynamic exclusion of previously acquired precursor ions was enabled at 18 s.

#### Data Processing

Raw MS files was managed by MaxQuant software (version 1.6.0.16), MS/MS-based peptide identification was carried out with the Andromeda search engine in MaxQuant, Andromeda uses a target-decoy approach to identify peptides and proteins at an FDR <1%. As a forward database, rat protein database from NCBI was used. A reverse database for the decoy search was generated automatically in MaxQuant. Enzyme specificity was set to “Trypsin,” and a minimum number of seven amino acids were required for peptide identification. Default settings were used for variable and fixed modifications [variable modification, acetylation (Protein-N terminus) and oxidation (methionine), fixed modification, carbamidomethylation]. A label-free, intensity-based absolute quantification (iBAQ) approach was used to calculate protein quantification based on the area under the curve (AUC) of precursor ions. The fraction of total (FOT) was used to represent the normalized abundance of a protein across experiments. The FOT was defined as a protein's iBAQ divided by the total iBAQ of all identified proteins in one experiment. The FOT was further multiplied by 105 to obtain iFOT for the ease of representation. Missing values were substituted with zeros.

### Multinomial Logistic Regression Model

Multinomial logistic regression is a multiclass linear classification method commonly used to classify multiclass categorical variables (22). In this study, drug treatments were regarded as dependent variables. The multinomial logistic regression model was implemented, using the Python Sklearn library v. 0.19.1 (23), to predict the sample categories. Initially, for each single-omics screening process, the data were stratified into training (80%) and testing (20%) data sets. A stratified sampling strategy, based on treatment, was used to determine the training–testing split, to obtain homogeneous subgroups. A mixed-effects modeling approach, using the omics data as fixed effects, was used; the characteristics of the rats, and of their organs, were used as random effects, because random effects accounted for variations between the rats and organs that might affect the response. “L2” regularization was used to prevent over-fitting: regularization strength was tuned through five-fold cross-validation. To evaluate feature selection robustness, the modeling process was repeated five times. In each iteration, the top features (500 for RNA-seq and 100 for protein data), ranked in order of their absolute coefficients in predicting each of the drug combinations (six combinations for RNA-seq and seven for protein data), were summarized into non-redundant sets (comprising 3,568 elements for RNA-seq and 1,157 elements for protein data). The resulting feature sets are thought to be highly associated with the drug treatment, and less related to the variables that are not the main focus; the selected feature sets were therefore used in downstream analysis. Pearson correlation analysis, and complete linkage hierarchical clustering analysis, were implemented to evaluate the stability of the feature coefficients, based on their similarity among the five iterations.

### Weighted Gene Co-expression Network Analysis

A signed weighted gene co-expression network was constructed in each single-omics data set using the WGCNA R package (24). The adjacency matrix was first constructed by weighting the Pearson correlation coefficient of molecule pairs with an estimated power (β; 20 for RNA-seq and 16 for protein data), which preserves continuous intrinsic connections without defining hard thresholds. Next, the adjacency matrix was transformed into a topological overlap matrix (TOM), providing the proximity measure of network interconnectedness. This matrix (1-TOM) was used as the input for downstream average linkage hierarchical clustering. Co-expression models were defined as the branches cut dynamically from the hierarchical tree. The co-expression pattern of a module was represented by its eigengene, also known as the first principal component. The intramodular hub nodes in a given module were selected based on their correlation with its eigengene. Sub-networks consisting of hub nodes and edges with strong connections were visualized using Cytoscape (25); hub nodes were ranked based on their connectivity within the sub-networks.

### Functional Enrichment Analysis

The ClusterProfiler package was used to implement functional enrichment analysis and visualization, based on the Gene Ontology (GO) and Kyoto Encyclopedia of Genes and Genomes (KEGG) databases (26). The biological functions related most significantly to high-altitude adaptation in the enrichment analysis were selected for visualization.

### Statistical Analysis

Data are presented as mean ± standard error. Between-group differences were analyzed using Student's *t*-tests or one-way ANOVA, followed by a Fisher's Least Significant Difference test. *P* < 0.05 was considered statistically significant.

## Results

### Validation of the HAPH Model

**Figure 1:**
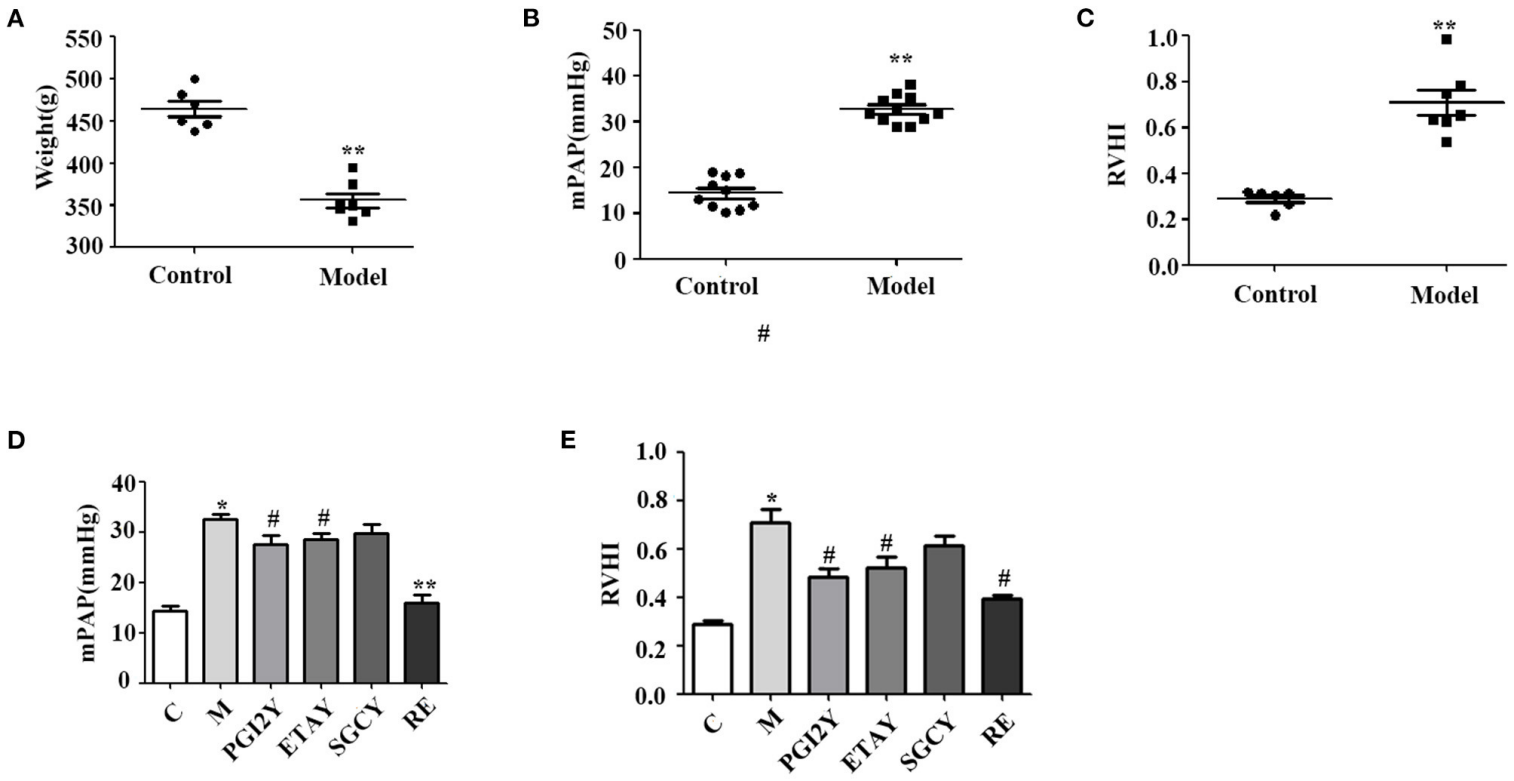
Establishment of the high-altitude pulmonary hypertension (HAPH) rat model, and the effects of the targeted drugs and reoxygenation on high-altitude pulmonary hypertension. **(A)** Weight. **(B)** Mean pulmonary artery pressure (mPAP). **(C)** Right ventricular hypertrophy index: RVHI = RV/(LV + IVS). **(D)** mPAP and **(E)** RVHI after treatment. C, control; M, model group; PGI2Y, selexipag treatment group; ETAY, macitentan treatment group; SGCY, riociguat treatment group; RE, reoxygenation group. Data represent mean ± SD. Student's *t*-tests: *P < 0.05 vs. control, ^#^*P* < 0.05 vs. M; **P < 0.0001 vs. M.

In the group exposed to hypobaric hypoxic conditions for 21 days, body weight decreased significantly from 463.83 to 355.57 g (Figure 1A, *P* < 0.0001). With prolonged hypoxia, mPAP increased gradually, until it was ~3 times higher in the hypoxic rats (Figure 1B, *P* < 0.0001). RVHI was significantly higher in rats kept under hypoxia (Figure 1C, *P* < 0.0001). These results indicate that the model of HAPH was successful.

### Responses of HAPH to Treatments

mPAP was significantly higher in the model rats. However, it decreased following selexipag, macitentan, and reoxygenation treatment (Figure 1D) and declined non-significantly following riociguat treatment. Because HAPH causes increased pulmonary artery pressure, it leads directly to RV hypertrophy. Therefore, RVHI increased significantly in group M, whereas it decreased in the selexipag, macitentan, and reoxygenation groups. Riociguat did not decrease this indicator (Figure 1E).

### Organ- and Treatment-Specific Co-expression Modules Identified *via* Mixed-Effects Modeling

**Figure 2:**
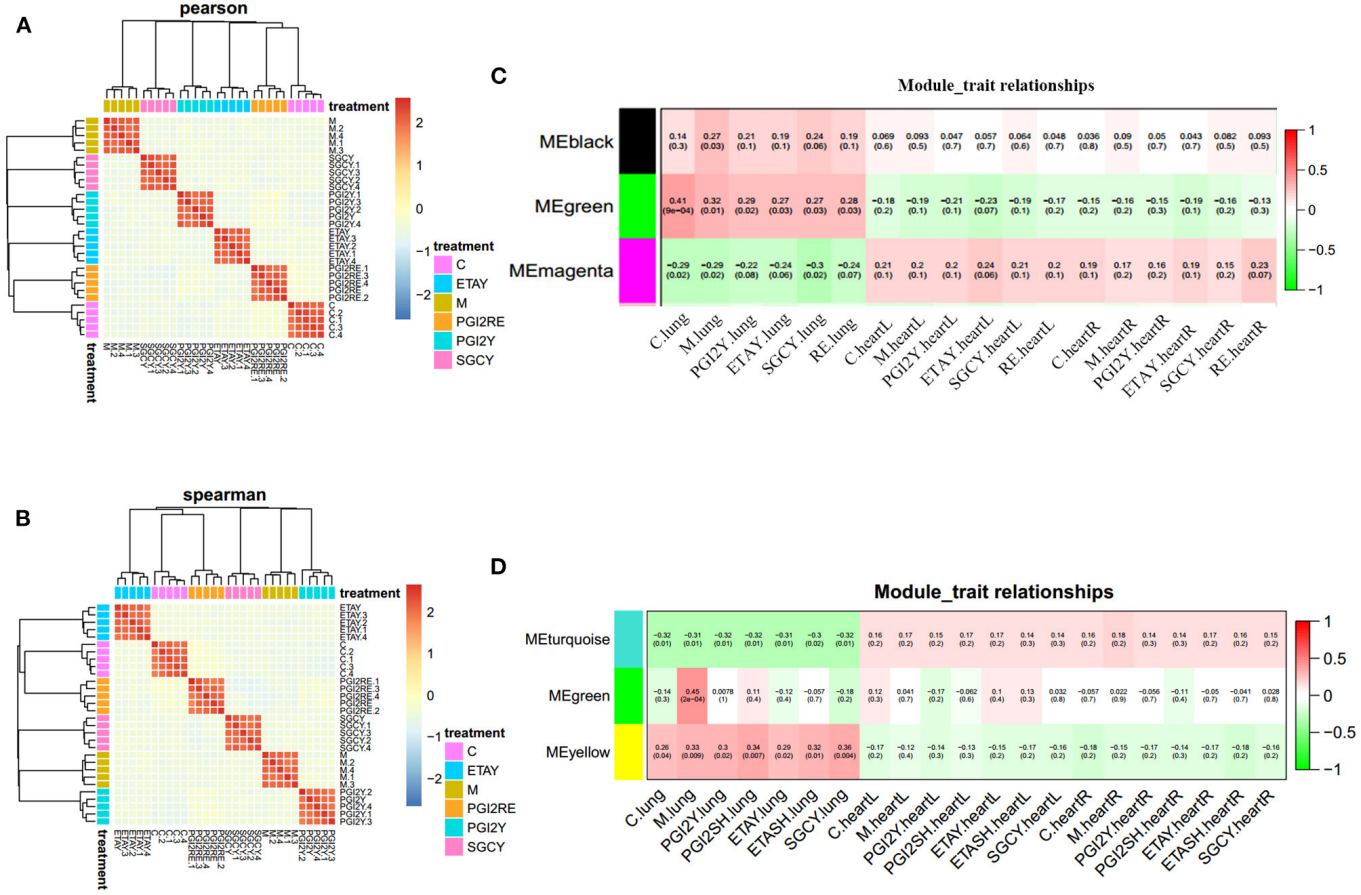
Feature selection robustness validation, and identification of co-expression modules. Correlation heatmap and hierarchical clustering tree of the feature coefficients in the drug-treatment classification models (with random sub-sampling) of **(A)** RNA-seq data and **(B)** protein data. The color reflects the Pearson correlation coefficient. Organ-specific co-expression modules identified in **(C)** RNA-seq data and **(D)** protein data, using Weighted Gene Co-expression Network Analysis, and their correlation to sample category. The Pearson correlation is listed above its associated *P*-value in each square. The color of each square corresponds to the correlation (red, positive; green, negative; white, no correlation). The black and green modules in **(C)** represent up regulation in the lungs. The magenta module in **(C)** represents upregulation in heart. The turquoise module in **(D)** represents unique in heart. The yellow module in **(D)** stands for unique in lung. The green module in **(D)** represents sharing in the heart and lung.

To avoid statistical noise, we use a mixed-effects model that uses RNA-seq data to model treatment effects. We use a model in which some of the genes can be used to accurately distinguish a sample from each treatment. As a result, we selected 3,568 genes and 1,157 proteins. In each model, the weights of these genes provide drug prediction vectors, which were used for correlation analysis. The drug treatments were highly correlated, and their effects were almost identical. This modeling method is stable, highly repeatable, and reliably identifies genes. The correlation heatmap and hierarchical clustering tree of the feature coefficients from the drug-treatment classification models (with random sub-sampling), for the RNA-seq data (Figure 2A) and protein data (Figure 2B), are shown; the color reflects the Pearson correlation coefficient.

We used WGCNA to obtain a co-expression network of known tissue-specific functional modules and selected the three co-expression modules most related to the organ sample types (Figures 2C,D): the different organs clearly have different molecular modules, and the LV and RV show consistent trends. For the RNA-Seq data, the black and green modules were most significantly correlated with the lungs. The magenta module was correlated with the heart. For the proteomics data, the turquoise module had the most significant correlation with the heart; the green module was correlated with the heart and lungs. The yellow module was correlated only to the lungs. The gene significance in these modules was imported into Cytoscape software to construct at the WGCN. MCODE was applied to filter the network module and select hub genes.

### RNA-seq Analysis Identified the Key Pathways Altered in HAPH and the Treatment Conditions

**Figure 3:**
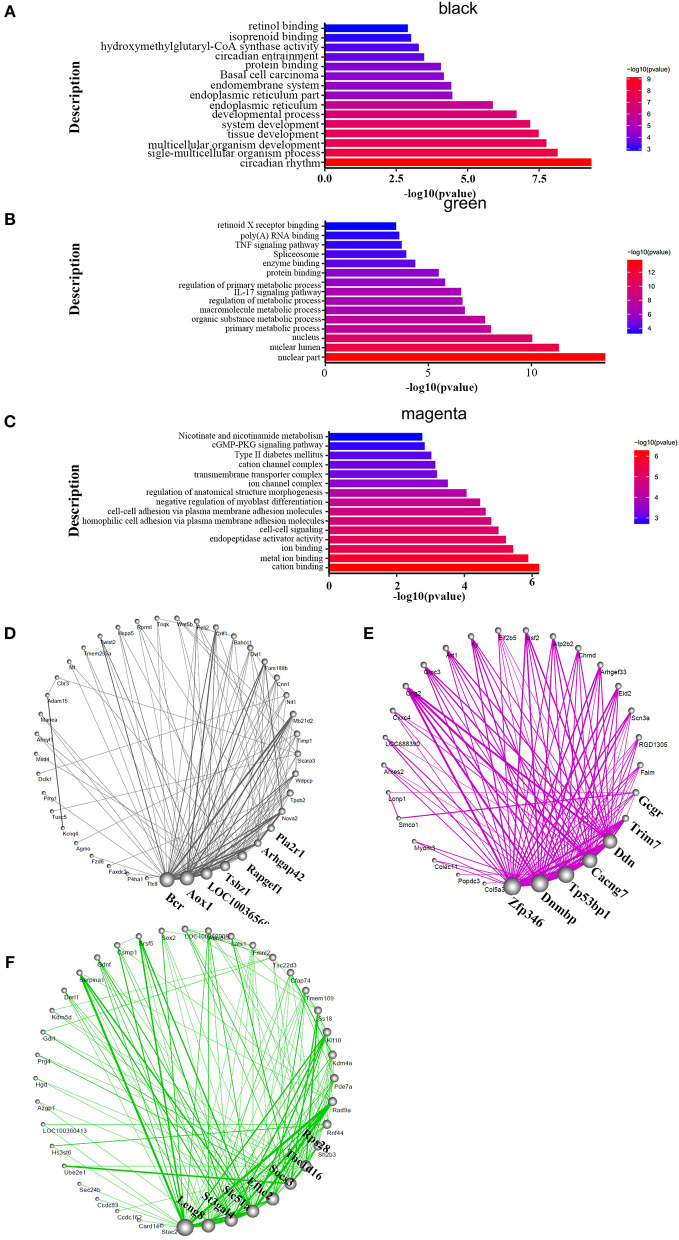
Functional enrichment analysis and hub molecular identification of co-expression networks based on RNA-seq data. **(A–C)** Biological functions enriched in organ-specific modules. Bar length indicates the –log_10_ of the *P*-value from the Fisher's exact test. Edge width represents connectivity strength. **(D–F)** Organ-specific co-expression hub networks. Edge width represents connectivity strength. Node size represents the level of hubness.

Genes act in a coordinated manner to carry out their biological functions. Pathway analysis helps to better understand the biological function of genes. In order to confirm the potential pathway in high-altitude pulmonary hypertension and administration of targeted drugs, the KEGG analyses were performed. Therefore, we identified the key pathways altered in HAPH and affected by the treatments. GO and KEGG functional enrichment analysis for each module, based on RNA-seq data, revealed many molecular pathways that may be associated with HAPH and its related adaptive mechanism. GO-enrichment analysis revealed that, among the enriched differentially expressed genes were those involved in metabolic regulation, transcription, and translation. The pathways were mainly enriched in ATP, gtpase activity, transcription factor, unfolded protein reaction, Wnt, Notch, Apelin, hemoglobin, oxygenase active, the rRNA processing metabolism, while the mitochondria promotes apoptosis in the black and green modules (Figures 3A,B). The enriched pathways are those involved in metabolic process such as retinol metabolism, tyrosine metabolism and butanoate metabolism; regulation of blood pressure; myoblast proliferation and differentiation; adrenergic signaling in cardiomyocytes; mitochondria and Wnt in the magenta module (Figure 3C). The detailed informations of the biological processes (GO_BP), cell components (GO_CC), molecular functions (GO_MF), and pathways enriched in kegg (KEGG) enriched in the GO database by different modules are shown in the Supplementary Table 1 based on the Figures 3A–C. We selected the core molecule in each module, to form the core gene sub-networks (Figures 3D–F; the color indicates the original module): the gene at the bottom of the figure is the most important, and importance decreases in a counterclockwise direction. The five most important genes (by module) were Bcr, Aox1, LOC100365697, Tshz1, and Rapgef1 (black module); Leng8, St3gal4, Slc51a, Efhc2, and Socs3 (green module); and Zfp346, Dnmbp, TP53bp1, Cacng7, and Ddn (magenta module). After selecting the hub genes, we further analyzed their effects by tissue, treatment, and organizational modules (Supplementary Figure 1): the radar charts each represent the effects of a molecule in an organization.

We also concluded that different modules may use some key pathways to extent. In the HIF-1 signaling pathway (Supplementary Figure 2), we found that the Cdkn1a, Vhl, Timp1, and Angpt2 genes were included in the black module. Eno4, Pfkfb3, Hmox1, Slc2a1, Rbx1, Rela, and Camk2g were shown in green module, while Hk1 was in magenta module. Referring to the TGF-β pathway (Supplementary Figure 3). LOC103691556, Bmp2, and Cul1 were included in the black module. Smad3, Acvr1b, Bmpr1b, Rbx1, and Thbs1 were chosen in green module. In magenta module, genes were Pitx2, Smad9, and Zfyve9. Regardless of the familiar pathways above, we also screened the Wnt signaling pathway (Supplementary Figure 4), Plcb1, Dkk2, Fzd3, Wnt5b, Dvl1, Wnt9a, Porcn, Fzd6, and Cul1were highlighted in black module. Apc, Rbx1, Notum, Prkacb, Vangl2, Smad3, Tcf7l2, and Camk2g were picked by the green module. However, the genes were Sfrp5, pc2, Ccnd2, Wnt9b, Dvl3, Nlk, Prickle3, Sfrp1, Cacybp, Sfrp2, and Cxxc4.

### Differential Protein Expression Was Observed in HAPH and Under the Treatments

**Figure 4:**
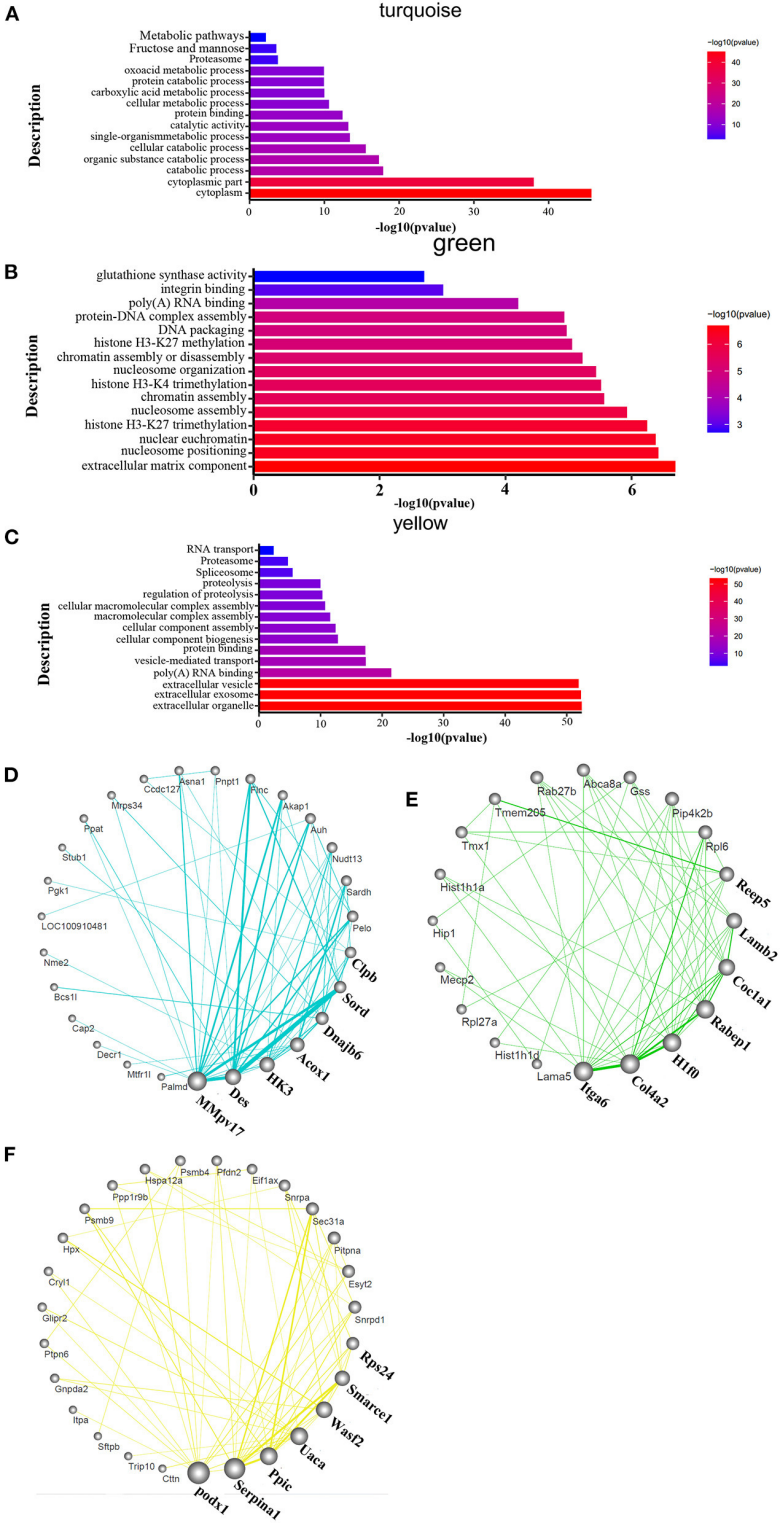
Functional enrichment analysis and hub molecular identification of co-expression networks based on protein data. **(A–C)** Biological functions enriched in organ-specific modules; bar length indicates the –log_10_ of the *P*-value from the Fisher's exact test. **(D–F)** Organ-specific co-expression hub networks. Edge width represents connectivity strength. Node size represents the level of hubness.

To identify the related signaling pathways and biological processes, we used GO analysis of the “biological process” category, and applied canonical pathway analysis to the turquoise, green, and yellow modules. The results show that pathways were mainly enriched in the processes of catabolism such as purine metabolism, D-Glutamine, and D-glutamate metabolism, fatty acid metabolism, oxoacid metabolic process and fructose and mannose metabolism; catalytic activity such as organic substance catabolic process and cellular catabolic process; protein binding; carboxylic acid metabolism; fructose and mannose metabolism in the turquoise module (Figure 4A). The green module was mostly enriched in the processes of extracellular matrix components, nucleosome localization, histone H3-K4 trimethylation, histone H3-K27 trimethylation, and glutathione synthase activity in the green module (Figure 4B). Processes related to vesicles, exosomes, cell assembly, proteolysis, proteasome, and RNA transport were enriched only in the yellow module (Figure 4C). The detailed informations of the biological processes (GO_BP), cell components (GO_CC), molecular functions (GO_MF), and pathways enriched in kegg (KEGG) enriched in the GO database by different modules are shown in the Supplementary Table 2 based on the Figures 4A–C. The organ-specific co-expression hub networks are shown in Figures 4D–F, and the relative expression levels of the organ-specific hub genes in the drug-treated corresponding organs are shown in Supplementary Figure 5.

### Integrated Multi-Omics Analysis to Verify Key Genes

The differential gene and protein expression data was used to integrate and correlate the multiple omics analyses. We compared the core genes of the modules that exhibited the same trends in RNA and protein expression, and comprehensively considered the importance of the RNA and corresponding proteins and their related genes in their respective omics modules. We then selected the following genes as the most important genes from the two sets of corresponding modules: Serpina1, Ccar2, Rps28, Pxn, Enoph1, Sec24b, S100a8, Glod4, Emd, U2af2, Ephx2, Cryz, Pter, Chordc1, Nt5c2, LOC102550385, Acy1, Cops2, Cmc1, and Usp9x. These represent RNA and proteins that are highly consistently associated with the RNA and protein datasets, which are important in both the genomics and proteomics analyses, and that may play roles in gene transcription and translation.

**Figure 5:**
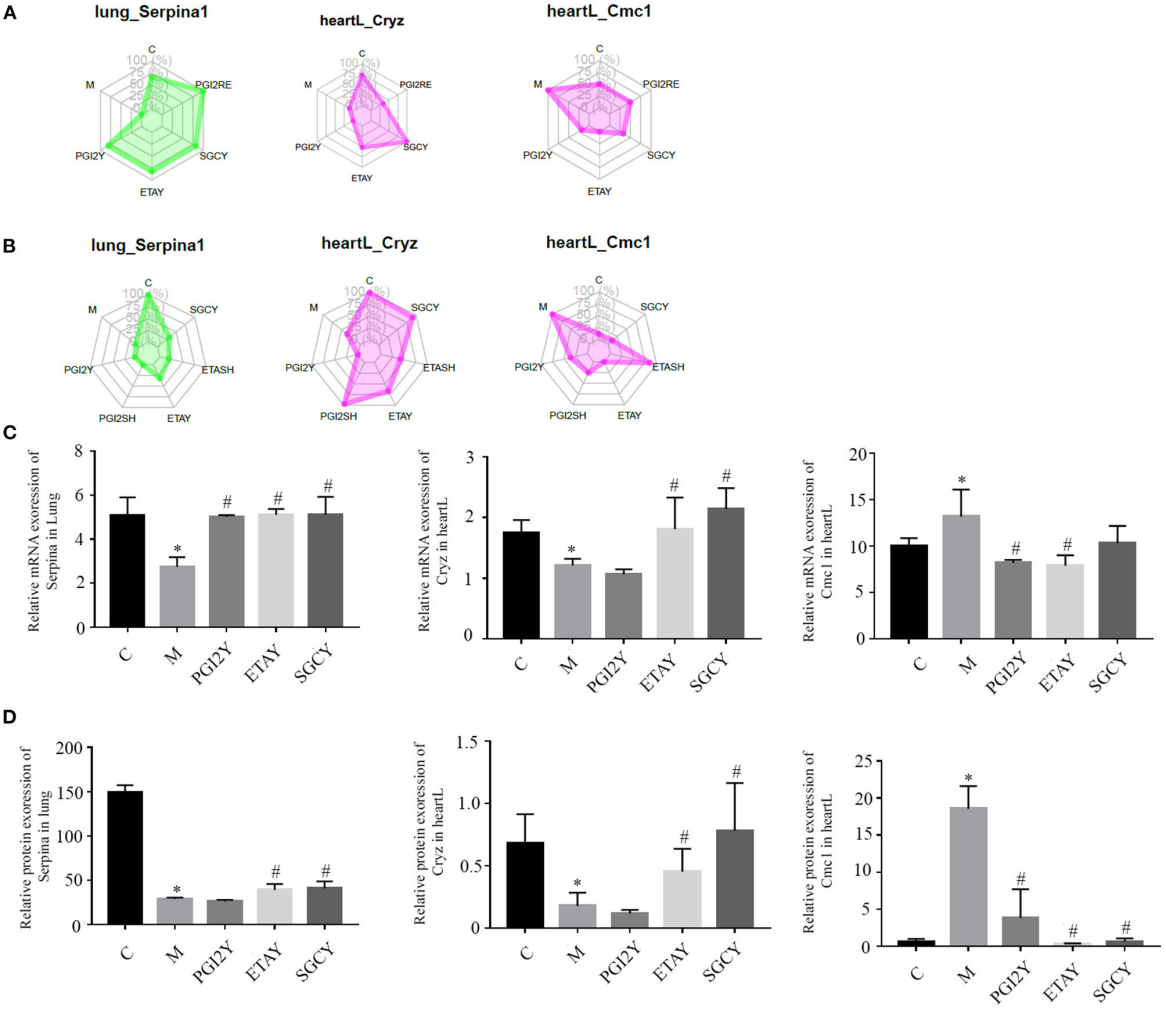
Hub molecules with consistent co-expression patterns in the multi-omics datasets. Relative expression levels of Serpina1, Cryz, and Cmc1 in corresponding modules based on **(A)** RNA-seq data and **(B)** protein data. Relative expression of **(C)** mRNA and **(D)** protein, of Serpina1, Cryz, and Cmc1. heartL, the left heart. *P < 0.05 vs. control, ^#^*P* < 0.05 vs. M.

The drugs differed in efficacy at the level of the important genes. Some of the drugs differed in these core molecules, and their therapeutic effects may differ. The hub molecules with consistent co-expression patterns in the genomic and proteomic datasets are labeled in Figure 5.

## Discussion

In our rat HAPH model, selexipag, macitentan, and reoxygenation significantly reduced mPAP, and riociguat reduced it insignificantly. Reoxygenation most effectively reduced mPAP, almost to the levels observed under normoxia. Similar results were observed for RVHI. As for the guidelines for the diagnosis and treatment, patients with PH who are hypoxaemic should receive long-term O_2_ therapy (27). Similarly, Sime et al. (5) reported the the effectiveness of oxygen therapy for HAPH, hence we set the reoxygenation-treated group as the positive group. Considering the few researches available on the targeted drugs on HAPH, we applied the drugs for further exploration.

Bellaye et al. (28) showed that AdTGF-β1-induced pulmonary fibrosis in rats is accompanied by PH, however, macitentan mitigated the development of PH induced by reduced mPAP. Macitentan could also improve monocrotaline-induced pulmonary arterial hypertension hemodynamically and histopathologically (29). In addition, the effect of macitentan is more effective than bosentan (30). In European treatment guidelines, selexipag were recommended to use in patients with PH and WHO functional class (FC) II or III (31). In Honda's research, they induced a pulmonary arterial hypertension model in SD rats by injecting the vascular endothelial growth factor receptor antagonist Sugen 5416. They also exposed Fischer rats to hypoxic conditions to induce PH. Experimental results show that selexipag could greatly ameliorate the right ventricular systolic pressure and right ventricular hypertrophy in SD rats. In the article, the authors demonstrated how selexipag attenuated the proportion of lung vessels with occlusive lesions and the medial wall thickness of lung arteries. It also reduced RV hypertrophy and mortality caused by RV failure in the model Fischer rats (32). Interestingly, in this study all of the rats of the hypoxia model were returned to normoxia after hypoxia for 3 weeks including the rats taken selexipag. However, the rats in our study were under hypoxic conditions and selexipag treatment simultaneously. As a soluble guanylate cyclase stimulator, the effect of riociguat does not depend on the levels of NO in the body. It can increase the levels of cGMP in plasma alone or synergistically with NO, causing vasodilation and anti-remodeling effects. In a 10 years follow-up study, riociguat could improve pulmonary vascular resistance and cardiac index for up to 8 years, but failed to improve pulmonary arterial pressure (33). In a clinical trial of in patients with pulmonary hypertension caused by systolic left ventricular dysfunction, riociguat did not decrease the mean pulmonary artery pressure, yet it improved cardiac index as well as pulmonary and systemic vascular resistance (34). In our study, according to the effect of riociguat on HAPH, the recovery of mean pulmonary artery pressure was not observed. However, in another study of rats with induced PH by the vascular endothelial growth factor receptor antagonist SU5416 and hypoxia, the effect of riociguat was more effective than that of sildenafil on PH (35).

GO and KEGG functional enrichment analysis of the RNA-Seq and proteomics data sets revealed the organizational modules enriched in various pathways. There was little overlap in the differences in enriched categories identified using the RNA-Seq and proteomics data. However, three genes including Serpina1, Cryz, and CMC1 were consistently identified by the two data sets and should be further studied.

Using RNA-seq, we observed that the differentially expressed genes were involved in metabolic regulation, transcription, and translation. Proteomics studies the protein composition of cells, tissues, or organisms, and their responses to stimuli. Differential protein expression in tissue or blood samples reveals how proteins change during pathogenesis; identifying the corresponding genes and metabolites makes it possible to study disease pathogenesis, diagnosis, and treatment. Using proteomics, the turquoise module was enriched mainly in the processes of catabolism, catalytic activity, protein binding, carboxylic acid metabolism, and fructose and mannose metabolism. The RNA-Seq and proteomics data produced different key GO terms related to differential expression. Absolute transcript abundance is often poorly correlated with protein expression levels; for instance, cardiac disease gene expression profiles had a limited commonality at the transcriptome and proteome levels (36). Similarly, our integrated approach, combining transcript abundance and protein turnover in HAPH, supports the notion that both transcriptional and post-transcriptional mechanisms affect pathogenesis in complex diseases.

Based on GO and KEGG functional enrichment analysis, the organizational modules were enriched in different pathways. Gtpase activity, in the black module, is an example of this. Rho, a small monomeric G protein, has gptase activity, and belongs to the Ras superfamily of proteins that regulate cell growth, differentiation, and survival (37). Rho participates in PH pathogenesis by promoting pulmonary vasoconstriction and structural remodeling. Rho-kinase inhibitors can induce acute pulmonary vasodilation, prevent PH, and induce pulmonary vascular remodeling (38–40). During PH pathogenesis, transcription factors are involved in the mechanisms of PH and of targeted treatment.

The unfolded protein response (UPR) process is mediated by three transmembrane receptor proteins in the endoplasmic reticulum (41). Low-pressure and low-oxygen conditions induce this process (42). Both endoplasmic reticulum stress and the UPR may play important roles in PH pathogenesis (43, 44).

HIF-1 is a heterodimer of HIF-1a and HIF-1b. It is a key regulator of oxygen homeostasis, and it could accommodate the adaptive molecular response under hypoxic conditions (45). The mechanism of HIF-1 in hypoxia-induced pulmonary hypertension has been clarified. It participated in the pathophysiologic alterations of both smooth muscle and endothelial cell biology in patients with PH (46). Further, it promotes vascular post-injury remodeling in both pulmonary and systemic arteries. Finally, it resulted in the apoptosis of pulmonary arterial smooth muscle cells and alleviation of pulmonary vascular remodeling when suppressing HIF-1 (47).

In our RNA-Seq analysis, the Notch pathway was screened. Notch proteins are cell membrane receptors that mediate signaling between cells, and hence play an important role in cell-to-cell communication (48). In the vascular system, the Notch pathway is involved in vascular development, angiogenesis, and arteriovenous specification. All of the screened pathways were related to HAPH. These pathways provide new targets for the treatment of HAPH.

After screening, we identified the three most important genes, Serpina1, Cryz, and CMC1, using the multi-omics datasets. Serpina1 encodes a serine protease inhibitor whose targets include plasmin, elastase, thrombin, trypsin, chymotrypsin, and plasminogen activator. This protein is secreted in the liver, the bone marrow, by lymphocytic and monocytic cells in lymphoid tissue, and by the Paneth cells of the gut. Serpina1 is a major circulating antiprotease. Pathogenic mutations in SERPINA1 gene will lead to α1-antitrypsin deficiency (AATD). α1-antitrypsin (AAT) was first discovered in 1963, and it was related to hereditary emphysema (49). Under normal homeostatic conditions, AAT could prevent damage of the lung alveolar matrix by regulating the proteolysis of human leukocyte elastase. AAT is secreted into the blood plasma, but its primary site of action is the lung parenchyma, despite it was secreted into the plasma (50). Many studies have proved that the up-regulated expression of AAT in monocytes will prevent the protease destruction in the lung microenvironment. This process will be regulated by the bacterial endotoxin and/or early production of inflammatory mediators such as interleukin-1 (IL-1) and tumor necrosis factor a (TNF-a) in the lung. Chronic obstructive pulmonary disease (COPD), emphysema, PH, pulmonary fibrosis, and chronic liver disease are related to Serpina1 deficiency (49). Furthermore, Hou et al. (51) reported that the interacting effect between lncRNAs and mRNAs on the pathogenesis of PH, in which the mRNA of Serpina1 was included. In our study, we found that Serpina1 played an important role in HAPH, hence it is worthy of further study.

Cryz encodes the crystallin zeta. The z-crystallin was found at first in the lenses of guinea pig (52). Crystallins are separated into two classes: taxon-specific, or enzyme, and ubiquitous. Cryz encodes a taxon-specific crystallin with NADPH-dependent quinone reductase activity distinct from other known quinone reductases. Cryz protein has a potentially pivotal role in cancer, allowing cells to hijack or subjugate the acidity response mechanism, to increase their ability to resist oxidative stress and apoptosis, while fueling their glutamine-addicted metabolism. However, CryZ protein was firstly discovered for its ability to bind DNA in cellfree settings (53). Later, Curthoys' group provided strong evidence that CryZ is an mRNA-binding protein. In a renal cell model, CryZ stabilizes rat glutaminase (GLS) mRNA (54). This protein binds specifically to adenine-uracil-rich elements in 3′-UTR of mRNA, for example bcl-2 and it has been reported to act as trans-acting factors in the regulation of certain mRNAs (55, 56) When it binds to bcl-2 mRNA, it will enhance the stability and effect of bcl-2 mRNA. In the Qi report, the researchers conducted a genome-wide association (GWA) study on circulating resistin levels in European individuals. The results indicated that novel loci near the TYW3/CRYZ gene (1p31) was associated with resistin levels. The resistin-rising allele (C-allele) of TYW3/CRYZ SNP rs3931020 was associated with increased coronary heart disease risk (55).

CMC1 encodes C-X9-C motif containing 1, which interacts and instantly stabilizes the early COX1–COX14–COA3 complex. CMC1 is regarded by some to be a COX1 chaperone. In a CMC1-knockout cell line, COX1 was able to synthesize normally, whereas mitochondrial respiratory chain complex IV (CIV) activity decreased (57), due to the instability of the newly synthetized COX1. As it is known, in the chronic thromboembolic pulmonary hypertension model, the impaired mitochondrial respiratory function participated in the development of right ventricular dysfunction. In addition, in the mitochondria containing 30–40% of the heart, CMC1 plays a vital role in the rat HAPH model. The drugs had different effects on these three core molecules; some of these effects may indicate therapeutic benefits. The three genes were the first engaged in the mechanism of pulmonary hypertension.

RNA-Seq and proteomics methods can effectively reveal genes or proteins related to hypobaric hypoxia-induced pulmonary hypertension. Using the GO function and KEGG pathway enrichment analysis of differentially expressed genes, we identified multiple pathways related to HAPH, and that responded to the targeted drugs. HAPH is a complex disease. Our findings show that reoxygenation and drug therapy can rescue abnormal gene expression, and restore affected pathways, in rats under simulated high-altitude hypoxia, thereby playing a role in myocardial protection.

## Conclusion

Selexipag, macitentan, and reoxygenation significantly attenuated the rat HAPH model, and riociguat had a weaker effect. Differentially expressed genes were involved in metabolic regulation, transcription, and translation. Certain proteins were affected by HAPH and by the treatments. We screened the key genes, Serpina1, Cryz, and Cmc1, using a multi-omics approach. These findings may provide a better understanding of the molecular mechanisms involved in hypoxia and may provide new therapeutic targets.

## Data Availability Statement

The raw data supporting the conclusions of this article will be made available by the authors, without undue reservation.

## Ethics Statement

The animal study was reviewed and approved by the Animal Ethics Committee of the Chinese PLA General Hospital.

## Author Contributions

CL and KH took responsibility for the integrity of the data, the accuracy of the data analysis, and designed the concept and obtained funding. XX, HL, and QW drafted of the manuscript, executed the experiments, did statistical analysis. XL, YS, GG, and YC carried out the data collection. All authors read and approved the final manuscript. All authors contributed to the article and approved the submitted version.

## Funding

This study was supported by the National Key Technologies R&D Program for New Drugs of China (Grant Number 2018ZX09J18109-004) and National Key R&D Program for Precision Medicine (Grant Number 2017YFC0908400).

## Conflict of Interest

The authors declare that the research was conducted in the absence of any commercial or financial relationships that could be construed as a potential conflict of interest.

## Publisher's Note

All claims expressed in this article are solely those of the authors and do not necessarily represent those of their affiliated organizations, or those of the publisher, the editors and the reviewers. Any product that may be evaluated in this article, or claim that may be made by its manufacturer, is not guaranteed or endorsed by the publisher.

## Abbreviations

HAPH: high-altitude pulmonary hypertension
NO: nitric oxide
sGC: soluble guanylate cyclase
cGMP: cyclic guanosine monophosphate
PGI2Y: selexipag-treated group
ETAY: macitentan-treated group
SGCY: riociguat-treated group
RE: reoxygenation group
LV: left ventricle
RV: right ventricle
mPAP: mean pulmonary arterial pressure
WGCNA: Weighted Gene Co-expression Network Analysis
TOM: topological overlap matrix
GO: Gene Ontology
KEGG: Kyoto Encyclopedia of Genes and Genomes
DTT: dithiothreitol
IAA: iodoacetamide
iBAQ: intensity-based absolute quantification
AUC: area under the curve
FOT: fraction of total
IL-1: interleukin-1
TNF-a: necrosis factor a
COPD: chronic obstructive pulmonary disease
GLS: glutaminase.

## References

[1] Kovacs G Dumitrescu D Barner A Greiner S Grunig E Hager A. Definition, clinical classification and initial diagnosis of pulmonary hypertension: updated recommendations from the Cologne Consensus Conference 2018. Int J Cardiol. (2018) 272S:11–9. 10.1016/j.ijcard.2018.08.083 30219257

[2] West JB. IX World Congress on high altitude medicine and physiology, Taipei, Taiwan, November 3-6, 2012. High Alt Med Biol. (2012) 13:140. 10.1089/ham.2012.1331 22994510

[3] Boucly A Weatherald J Savale L Jais X Cottin V Prevot G. Risk assessment, prognosis and guideline implementation in pulmonary arterial hypertension. Eur Respir J. (2017) 50:1700889. 10.1183/13993003.00889-2017 28775050

[4] Eichstaedt CA Antao T Cardona A Pagani L Kivisild T Mormina M. Genetic and phenotypic differentiation of an Andean intermediate altitude population. Physiol Rep. (2015) 3:e12376. 10.14814/phy2.12376 25948820 PMC4463816

[5] Sime F Penaloza D Ruiz L. Bradycardia, increased cardiac output, and reversal of pulmonary hypertension in altitude natives living at sea level. Br Heart J. (1971) 33:647–57. 10.1136/hrt.33.5.647 5115010 PMC487232

[6] Mirrakhimov AE Strohl KP. High-altitude pulmonary hypertension: an update on disease pathogenesis and management. Open Cardiovasc Med J. (2016) 10:19–27. 10.2174/1874192401610010019 27014374 PMC4780514

[7] Iglarz M Binkert C Morrison K Fischli W Gatfield J Treiber A. Pharmacology of Macitentan, an orally active tissue-targeting dual endothelin receptor antagonist. J Pharmacol Exp Ther. (2008) 327:736–45. 10.1124/jpet.108.142976 18780830

[8] Patel T McKeage K. Macitentan: first global approval. Drugs. (2014) 74:127–33. 10.1007/s40265-013-0156-6 24297706

[9] Pulido T Adzerikho I Channick RN Delcroix M Galie N Ghofrani HA. Macitentan and morbidity and mortality in pulmonary arterial hypertension. New Engl J Med. (2013) 369:809–18. 10.1056/NEJMoa1213917 23984728

[10] Khadka A Singh Brashier DB Tejus A Sharma AK. Macitentan: an important addition to the treatment of pulmonary arterial hypertension. J Pharmacol Pharmacother. (2015) 6:53–7. 10.4103/0976-500X.149151 25709357 PMC4319253

[11] Sitbon O Channick R Chin KM Frey A Gaine S Galie N. Selexipag for the treatment of pulmonary arterial hypertension. New Engl J Med. (2015) 373:2522–33. 10.1056/NEJMoa1503184 26699168

[12] Frost A Janmohamed M Fritz JS McConnell JW Poch D Fortin TA. Safety and tolerability of transition from inhaled treprostinil to oral selexipag in pulmonary arterial hypertension: results from the TRANSIT-1 study. J Heart Lung Transplant. (2019) 38:43–50. 10.1016/j.healun.2018.09.003 30391194

[13] Archer SL Michelakis ED. Phosphodiesterase type 5 inhibitors for pulmonary arterial hypertension. N Engl J Med. (2009) 361:1864–71. 10.1056/NEJMct0904473 19890129

[14] Wharton J Strange JW Moller GM Growcott EJ Ren X Franklyn AP. Antiproliferative effects of phosphodiesterase type 5 inhibition in human pulmonary artery cells. Am J Respir Crit Care Med. (2005) 172:105–13. 10.1164/rccm.200411-1587OC 15817798

[15] Nagendran J Archer SL Soliman D Gurtu V Moudgil R Haromy A. Phosphodiesterase type 5 is highly expressed in the hypertrophied human right ventricle, and acute inhibition of phosphodiesterase type 5 improves contractility. Circulation. (2007) 116:238–48. 10.1161/CIRCULATIONAHA.106.655266 17606845

[16] Ghofrani HA Galie N Grimminger F Grunig E Humbert M Jing ZC. Riociguat for the treatment of pulmonary arterial hypertension. N Engl J Med. (2013) 369:330–40. 10.1056/NEJMoa1209655 23883378

[17] Schermuly RT Janssen W Weissmann N Stasch JP Grimminger F Ghofrani HA. Riociguat for the treatment of pulmonary hypertension. Expert Opin Investig Drugs. (2011) 20:567–76. 10.1517/13543784.2011.565048 21391889

[18] Gao X Zhang Z Li X Li C Hao J Luo Y. Macitentan attenuates chronic mountain sickness in rats by regulating arginine and purine metabolism. J Proteome Res. (2020) 19:3302–14. 10.1021/acs.jproteome.0c00219 32640793

[19] Parkhomchuk D Borodina T Amstislavskiy V Banaru M Hallen L Krobitsch S. Transcriptome analysis by strand-specific sequencing of complementary DNA. Nucleic Acids Res. (2009) 37:e123. 10.1093/nar/gkp596 19620212 PMC2764448

[20] Bolstad BM Irizarry RA Astrand M Speed TP. A comparison of normalization methods for high density oligonucleotide array data based on variance and bias. Bioinformatics. (2003) 19:185–93. 10.1093/bioinformatics/19.2.185 12538238

[21] San Segundo E Tsanas A Gomez-Vilda P. Euclidean Distances as measures of speaker similarity including identical twin pairs: a forensic investigation using source and filter voice characteristics. Forensic Sci Int. (2017) 270:25–38. 10.1016/j.forsciint.2016.11.020 27912151 PMC5698260

[22] Böhning D. Multinomial logistic regression algorithm. Ann Inst Stat Math. (1992) 44:197–200. 10.1007/BF00048682

[23] Fabian P Gaël V Alexandre G Vincent M Bertrand T Olivier G. Scikit-learn: machine learning in Python. J Mach Learn Res. (2011) 12:2825–30.

[24] Langfelder P Horvath S. WGCNA: an R package for weighted correlation network analysis. BMC Bioinformatics. (2008) 9:559. 10.1186/1471-2105-9-559 19114008 PMC2631488

[25] Shannon P Markiel A Ozier O Baliga NS Wang JT Ramage D. Cytoscape: a software environment for integrated models of biomolecular interaction networks. Genome Res. (2003) 13:2498–504. 10.1101/gr.1239303 14597658 PMC403769

[26] Yu G Wang LG Han Y He QY. clusterProfiler: an R package for comparing biological themes among gene clusters. OMICS. (2012) 16:284–7. 10.1089/omi.2011.0118 22455463 PMC3339379

[27] Galie N Humbert M Vachiery JL Gibbs S Lang I Torbicki A. ESC/ERS Guidelines for the diagnosis and treatment of pulmonary hypertension: the Joint Task Force for the Diagnosis and Treatment of Pulmonary Hypertension of the European Society of Cardiology (ESC) and the European Respiratory Society (ERS): Endorsed by: Association for European Paediatric and Congenital Cardiology (AEPC), International Society for Heart and Lung Transplantation (ISHLT). Eur Heart J. (2016) 37:67–119. 10.1093/eurheartj/ehv317 26320113

[28] Bellaye PS Yanagihara T Granton E Sato S Shimbori C Upagupta C. Macitentan reduces progression of TGF-β1-induced pulmonary fibrosis and pulmonary hypertension. Eur Respir J. (2018) 52:1701857. 10.1183/13993003.01857-2017 29976656

[29] Kim KH Kim HK Chan SY Kim YJ Sohn DW. Hemodynamic and histopathologic benefits of early treatment with macitentan in a rat model of pulmonary arterial hypertension. Korean Circ J. (2018) 48:839–53. 10.4070/kcj.2017.0394 30088353 PMC6110709

[30] Iglarz M Landskroner K Bauer KY Vercauteren M Rey M Renault B. Comparison of Macitentan and Bosentan on right ventricular remodeling in a rat model of non-vasoreactive pulmonary hypertension. J Cardiovasc Pharmacol. (2015) 66:457–67. 10.1097/FJC.0000000000000296 26230396 PMC4632117

[31] Panagiotidou E Boutou A Pitsiou G. An evaluation of selexipag for the treatment of pulmonary hypertension. Expert Opin Pharmacother. (2021) 22:29–36. 10.1080/14656566.2020.1812579 32867545

[32] Honda Y Kosugi K Fuchikami C Kuramoto K Numakura Y Kuwano K. The selective PGI2 receptor agonist selexipag ameliorates Sugen 5416/hypoxia-induced pulmonary arterial hypertension in rats. PLoS One. (2020) 15:e0240692. 10.1371/journal.pone.0240692 33057388 PMC7561119

[33] Yang S Yang Y Zhang Y Kuang T Gong J Li J. Haemodynamic effects of riociguat in CTEPH and PAH: a 10-year observational study. ERJ Open Res. (2021) 7:00082–2021. 10.1183/23120541.00082-2021 34513985 PMC8419318

[34] Bonderman D Ghio S Felix SB Ghofrani HA Michelakis E Mitrovic V. Left ventricular systolic dysfunction associated with pulmonary hypertension Riociguat Trial Study, Riociguat for patients with pulmonary hypertension caused by systolic left ventricular dysfunction: a phase IIb double-blind, randomized, placebo-controlled, dose-ranging hemodynamic study. Circulation. (2013) 128:502–11. 10.1161/CIRCULATIONAHA.113.001458 23775260

[35] Lang M Kojonazarov B Tian X Kalymbetov A Weissmann N Grimminger F. The soluble guanylate cyclase stimulator riociguat ameliorates pulmonary hypertension induced by hypoxia and SU5416 in rats. PLoS One. (2012) 7:e43433. 10.1371/journal.pone.0043433 22912874 PMC3422306

[36] Foster DB Liu T Kammers K O'Meally R Yang N Papanicolaou KN. Integrated omic analysis of a guinea pig model of heart failure and sudden cardiac death. J Proteome Res. (2016) 15:3009–28. 10.1021/acs.jproteome.6b00149 27399916 PMC5779628

[37] Sakurada S Okamoto H Takuwa N Sugimoto N Takuwa Y. Rho activation in excitatory agonist-stimulated vascular smooth muscle. Am J Physiol Cell Physiol. (2001) 281:C571–C8. 10.1152/ajpcell.2001.281.2.C571 11443056

[38] Ishikura K Yamada N Ito M Ota S Nakamura M Isaka N. Beneficial acute effects of rho-kinase inhibitor in patients with pulmonary arterial hypertension. Circ J. (2006) 70:174–8. 10.1253/circj.70.174 16434811

[39] Sun XZ Li SY Tian XY Hong Z Li JX. Effect of Rho kinase inhibitor fasudil on the expression ET-1 and NO in rats with hypoxic pulmonary hypertension. Clin Hemorheol Microcirc. (2019) 71:3–8. 10.3233/CH-160232 29660902

[40] Do eZ Fukumoto Y Takaki A Tawara S Ohashi J Nakano M. Evidence for Rho-kinase activation in patients with pulmonary arterial hypertension. Circ J. (2009) 73:1731–9. 10.1253/circj.CJ-09-0135 19590140

[41] Kohno K. How transmembrane proteins sense endoplasmic reticulum stress. Antioxid Redox Signal. (2007) 9:2295–303. 10.1089/ars.2007.1819 17896870

[42] Jain K Suryakumar G Prasad R Singh SN Ganju L. Myocardial ER chaperone activation and protein degradation occurs due to synergistic, not individual, cold and hypoxic stress. Biochimie. (2013) 95:1897–908. 10.1016/j.biochi.2013.06.018 23816873

[43] Dromparis P Paulin R Stenson TH Haromy A Sutendra G Michelakis ED. Attenuating endoplasmic reticulum stress as a novel therapeutic strategy in pulmonary hypertension. Circulation. (2013) 127:115–25. 10.1161/CIRCULATIONAHA.112.133413 23149668

[44] Koyama M Furuhashi M Ishimura S Mita T Fuseya T Okazaki Y. Reduction of endoplasmic reticulum stress by 4-phenylbutyric acid prevents the development of hypoxia-induced pulmonary arterial hypertension. Am J Physiol Heart Circ Physiol. (2014) 306:H1314–H23. 10.1152/ajpheart.00869.2013 24610918

[45] Semenza GL. Regulation of oxygen homeostasis by hypoxia-inducible factor 1. Physiology (Bethesda). (2009) 24:97–106. 10.1152/physiol.00045.2008 19364912

[46] Semenza GL. Involvement of hypoxia-inducible factor 1 in pulmonary pathophysiology. Chest. (2005) 128:592S–4S. 10.1378/chest.128.6_suppl.592S 16373853

[47] Yan Y Shen Y Wang Y Li BB. Increased expression of hypoxia-inducible factor-1alpha in proliferating neointimal lesions in a rat model of pulmonary arterial hypertension. Am J Med Sci. (2013) 345:121–8. 10.1097/MAJ.0b013e31824cf5a2 22627259

[48] Artavanis-Tsakonas S Rand MD Lake RJ. Notch signaling: cell fate control and signal integration in development. Science. (1999) 284:770–6. 10.1126/science.284.5415.770 10221902

[49] Zhu W Li L Deng M Wang B Li M Ding G. Oxidation-resistant and thermostable forms of alpha-1 antitrypsin from *Escherichia coli* inclusion bodies. FEBS Open Bio. (2018) 8:1711–21. 10.1002/2211-5463.12515 30338221 PMC6168689

[50] Moraga F Janciauskiene S. Activation of primary human monocytes by the oxidized form of alpha1-antitrypsin. J Biol Chem. (2000) 275:7693–700. 10.1074/jbc.275.11.7693 10713080

[51] Hou S Chen D Liu J Chen S Zhang X Zhang Y. Profiling and molecular mechanism analysis of long non-coding RNAs and mRNAs in pulmonary arterial hypertension rat models. Front Pharmacol. (2021) 12:709816. 10.3389/fphar.2021.709816 34267668 PMC8277419

[52] Rodriguez IR Gonzalez P Zigler JS Borras T. A guinea-pig hereditary cataract contains a splice-site deletion in a crystallin gene. Biochim Biophys Acta. (1992) 1180:44–52. 10.1016/0925-4439(92)90025-I 1390943

[53] Kranthi BV Balasubramanian N Rangarajan PN. Isolation of a single-stranded DNA-binding protein from the methylotrophic yeast, *Pichia pastoris* and its identification as zeta crystallin. Nucleic Acids Res. (2006) 34:4060–8. 10.1093/nar/gkl577 16914438 PMC1557824

[54] Lulli M Nencioni D Papucci L Schiavone N. Zeta-crystallin: a moonlighting player in cancer. Cell Mol Life Sci. (2020) 77:965–76. 10.1007/s00018-019-03301-3 31563996 PMC11104887

[55] Qi Q Menzaghi C Smith S Liang L de Rekeneire N Garcia ME. Genome-wide association analysis identifies TYW3/CRYZ and NDST4 loci associated with circulating resistin levels. Hum Mol Genet. (2012) 21:4774–80. 10.1093/hmg/dds300 22843503 PMC3471394

[56] Lapucci A Lulli M Amedei A Papucci L Witort E Gesualdo FDi. zeta-Crystallin is a bcl-2 mRNA binding protein involved in bcl-2 overexpression in T-cell acute lymphocytic leukemia. FASEB J. (2010) 24:1852–65. 10.1096/fj.09-140459 20103721 PMC2874474

[57] Bourens M Barrientos A. A CMC1-knockout reveals translation-independent control of human mitochondrial complex IV biogenesis. EMBO Rep. (2017) 18:477–94. 10.15252/embr.201643103 28082314 PMC5331208

