# Supplementary material for: Novel Targets in a High-Altitude Pulmonary Hypertension Rat Model Based on RNA-seq and Proteomics
Source: Front Med (Lausanne). 2021 Nov 3;8:742436. doi: 10.3389/fmed.2021.742436 (PMC8595261; doi:10.3389/fmed.2021.742436)
Supplement: Supplementary file 1 [file Table_1.DOCX]

**Supplemental Figure Legends**

Supplemental figure 1. Functional enrichment analysis and hub molecular identification of co-expression networks based on RNA-seq data and protein data. Relative expression of the organ-specific hub gene in the drug-treated samples of corresponding organs. heartL: left ventricle ; heartR: right ventricle; c:control group; M: model group; PGI2RE: reoxygenation- treated group; SGCY: riociguat-treated group; ETAY: macitentan-treated group; PGI2Y: selexipag-treated group.

Supplemental figure 2. Different modules used the HIF-1 signaling pathway. Genes in black box represented the ones enriched in black module; genes in green box represented the ones enriched in green module; genes in magenta box represented the ones enriched in magenta module.

Supplemental figure 3. Different modules used the TGF-β signaling pathway. Genes in black box represented the ones enriched in black module; genes in green box represented the ones enriched in green module; genes in magenta box represented the ones enriched in magenta module.

Supplemental figure 4. Different modules used the Wnt signaling pathway. Genes in black box represented the ones enriched in black module; genes in green box represented the ones enriched in green module; genes in magenta box represented the ones enriched in magenta module.

Supplemental figure 5. Functional enrichment analysis and hub molecular identification of co-expression networks based on protein data. Relative expression of the organ-specific hub gene in the drug-treated samples of corresponding organs.
