# Supplementary figures and images for: Novel Targets in a High-Altitude Pulmonary Hypertension Rat Model Based on RNA-seq and Proteomics
Source: Front Med (Lausanne). 2021 Nov 3;8:742436. doi: 10.3389/fmed.2021.742436 (PMC8595261; doi:10.3389/fmed.2021.742436)

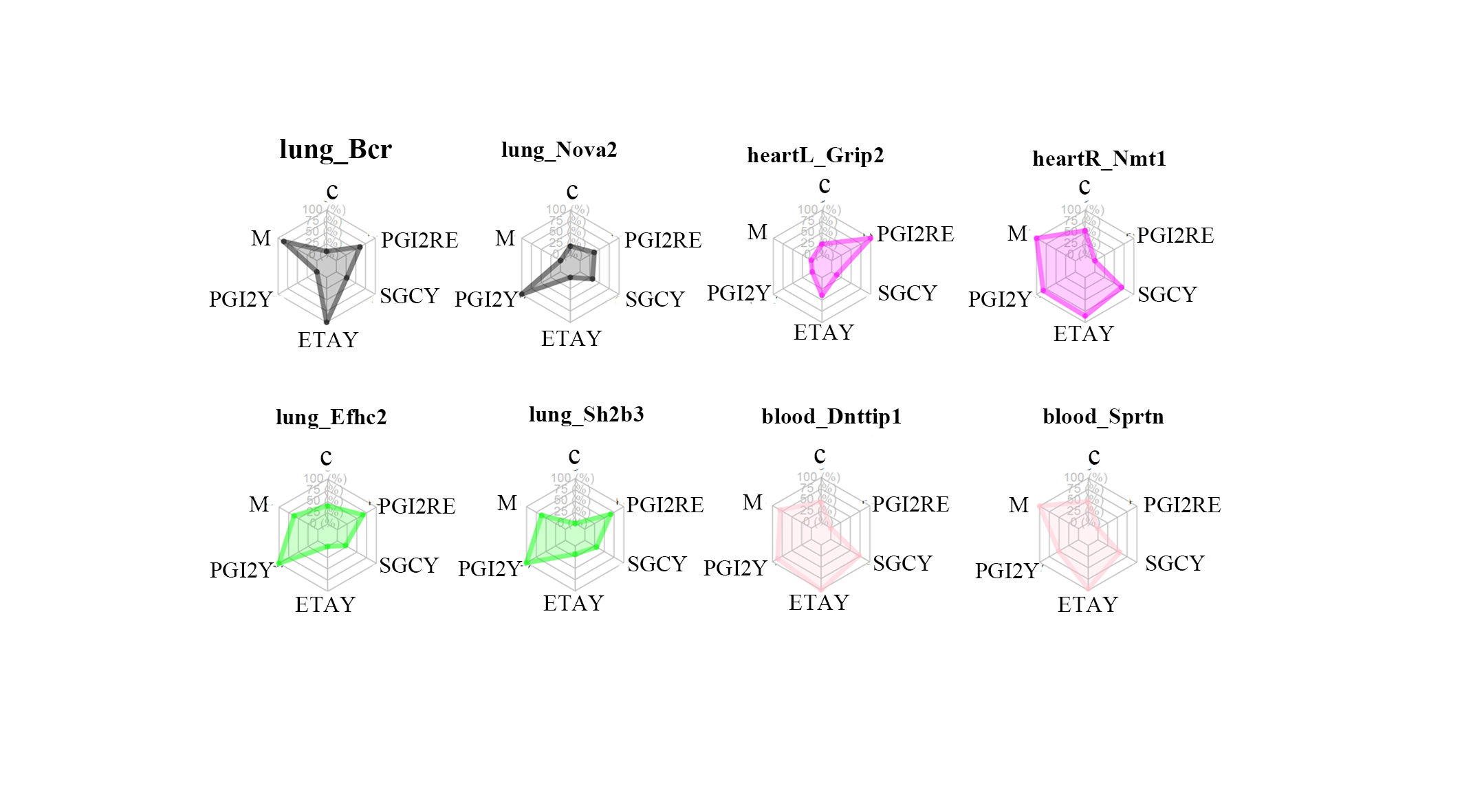

Supplement: Supplementary file 4 [file Image_1.TIF]

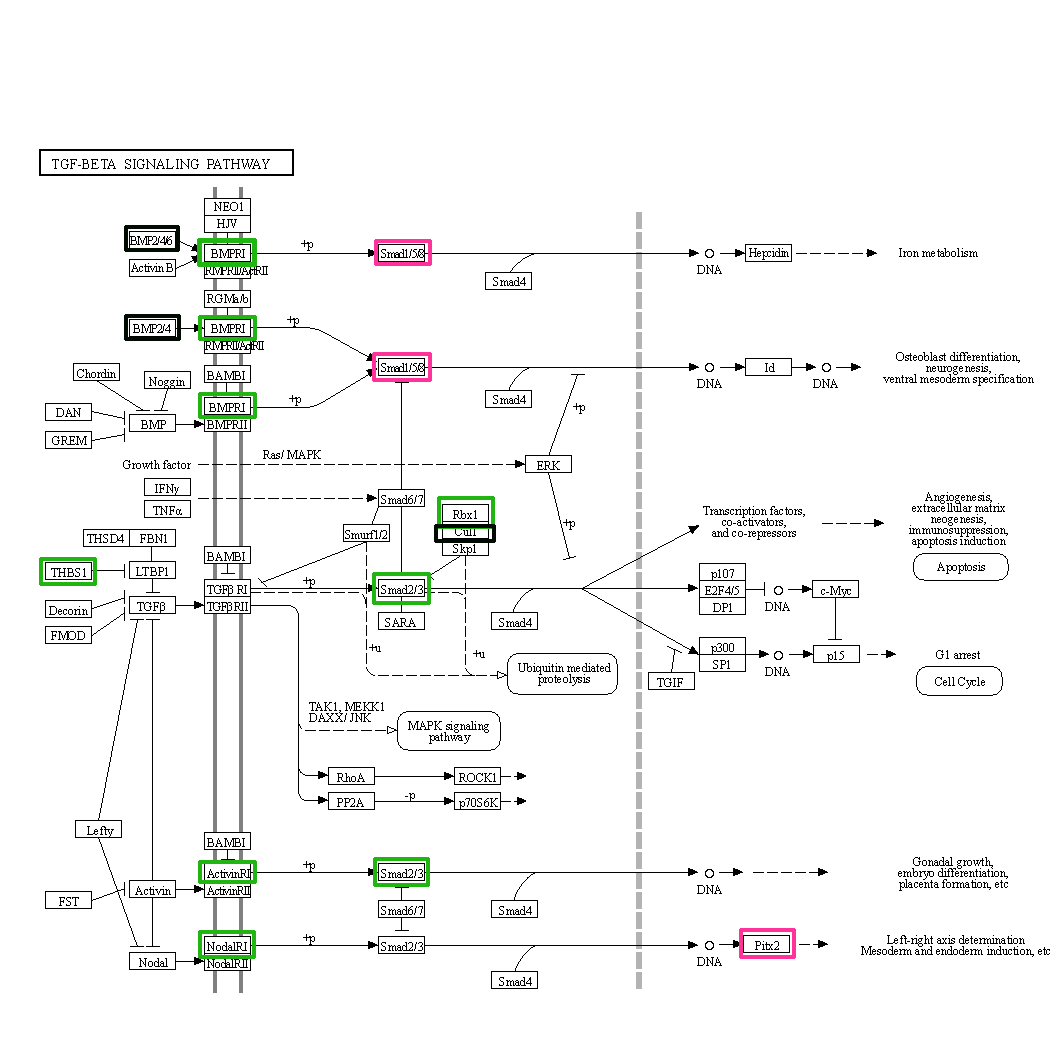

Supplement: Supplementary file 5 [file Image_2.TIF]

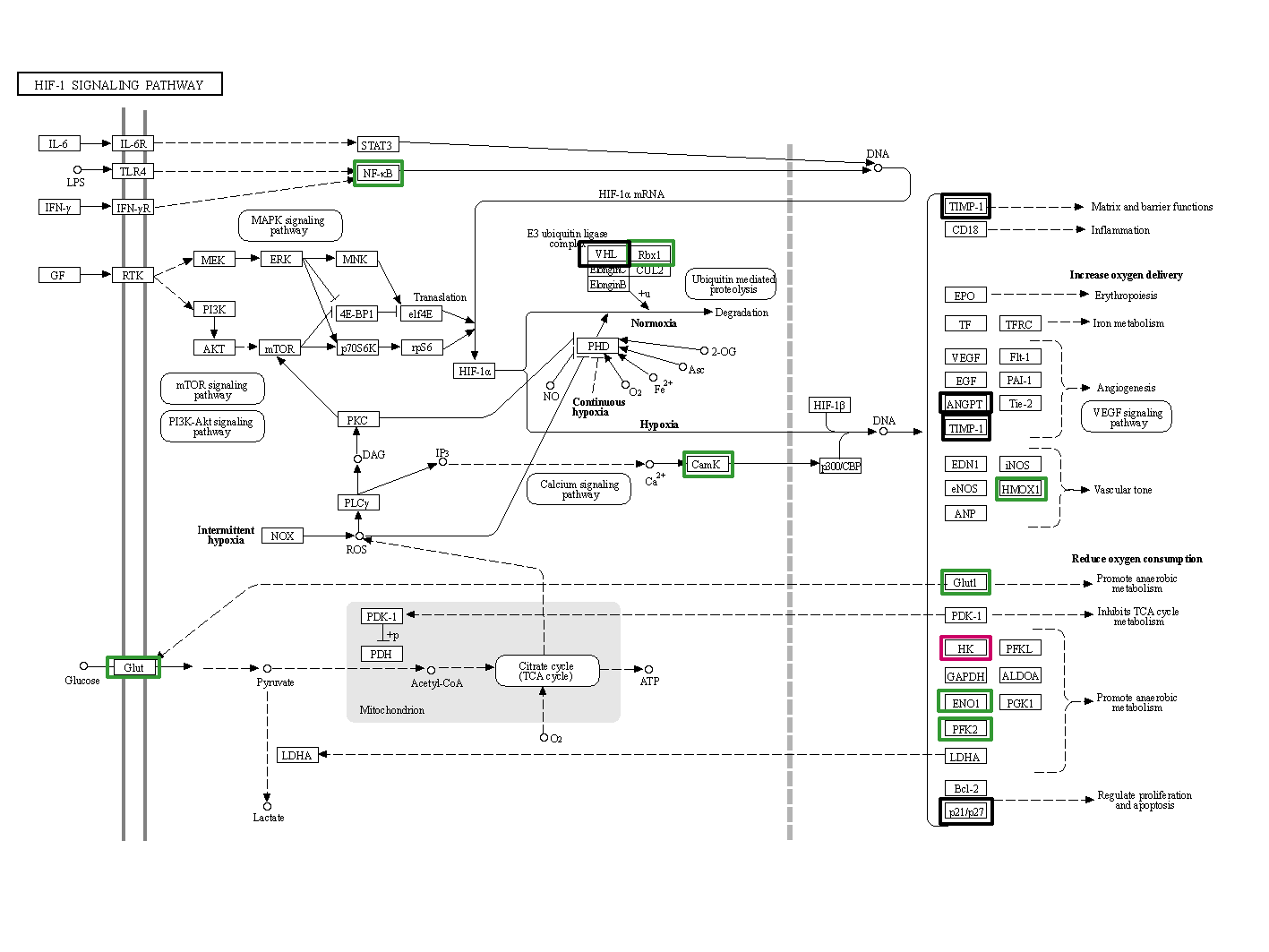

Supplement: Supplementary file 6 [file Image_3.TIF]

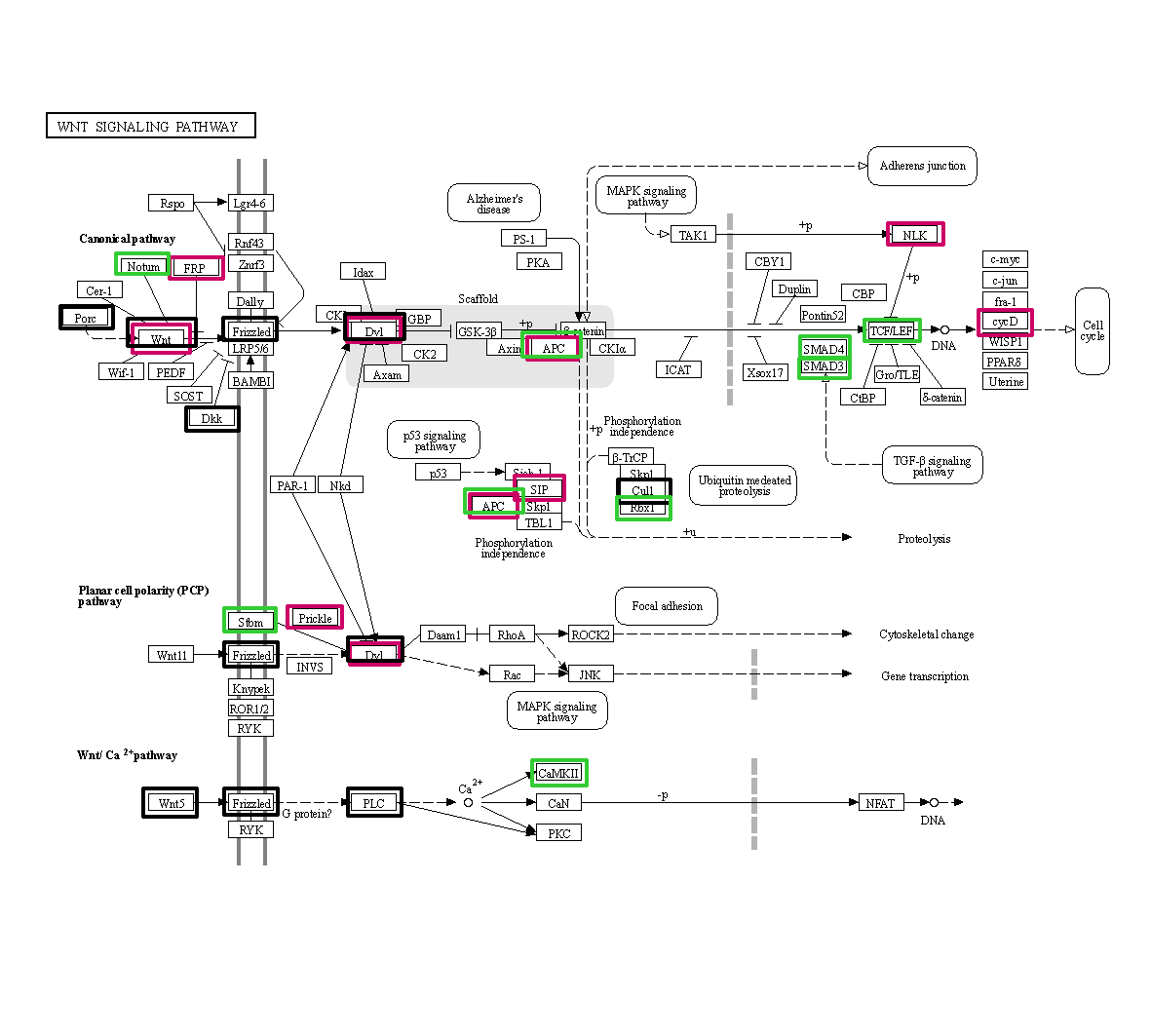

Supplement: Supplementary file 7 [file Image_4.TIF]

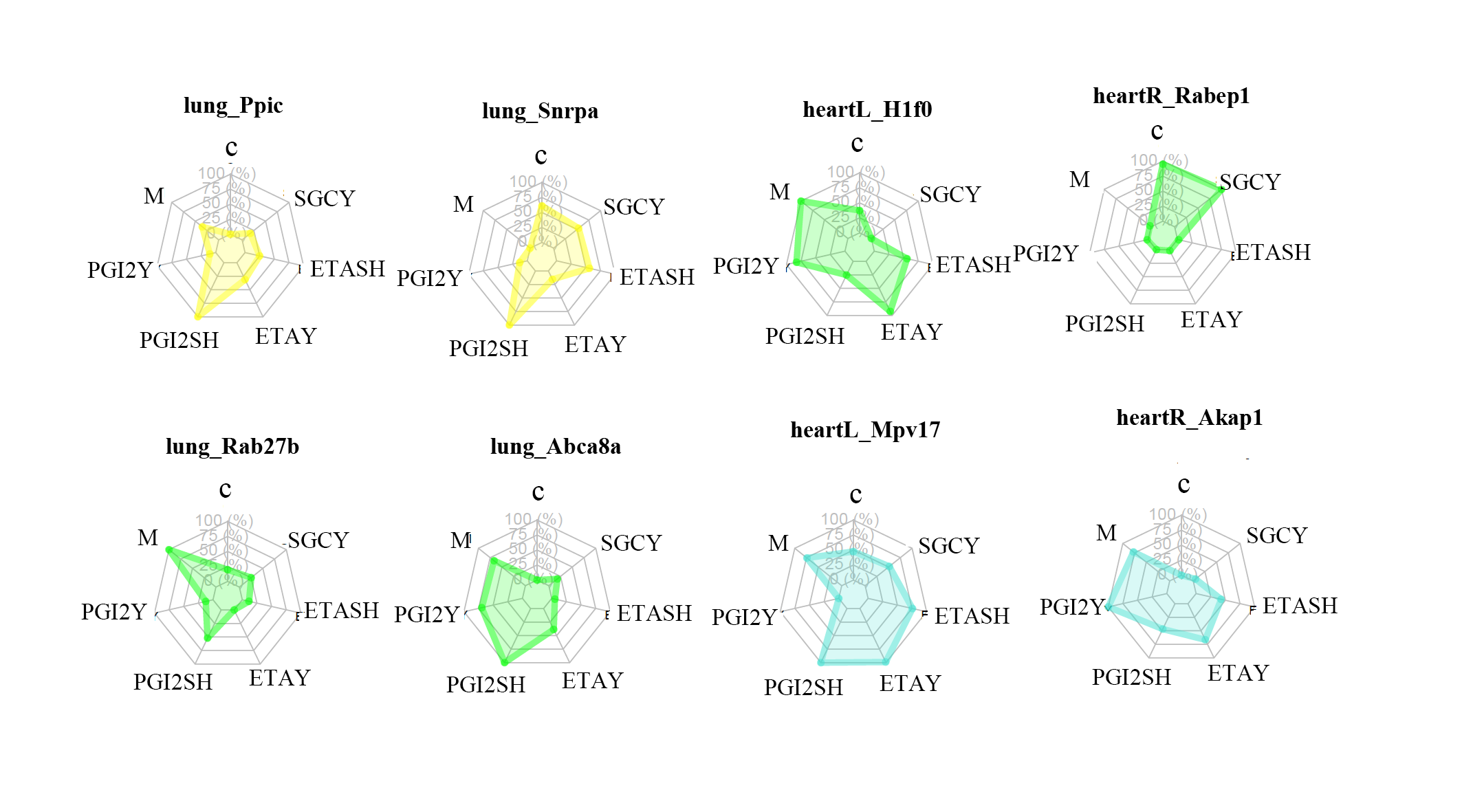

Supplement: Supplementary file 8 [file Image_5.TIF]
